# Dieulafoy lesion: two pediatric case reports

**DOI:** 10.1186/s13052-020-0814-8

**Published:** 2020-04-19

**Authors:** Giovanni Di Nardo, Gianluca Esposito, Angela Mauro, Letizia Zenzeri, Gian Paolo Ciccarelli, Andrea Catzola, Alessandro Rossi, Vito Domenico Corleto

**Affiliations:** 1 Chair of Pediatrics, NESMOS Department, Faculty of Medicine and Psychology, Sapienza University of Rome, Sant’Andrea University Hospital, Rome, Italy; 2 Gastrointestinal Endoscopy Unit, Department of Medical-Surgical Sciences and Translational Medicine, Sant’Andrea Hospital, Sapienza University of Rome, Rome, Italy; 3 Pediatric Emergency Unit, AORN Santobono-Pausilipon, Naples, Italy; 4 Department of Translational Medical Science, Section of Pediatrics, University of Naples “Federico II”, Naples, Italy

**Keywords:** Children, Dieulafoy, Gastrointestinal bleeding, Banding, Hemostasis, Clipping

## Abstract

**Background:**

Massive gastrointestinal bleeding in children is uncommon. Dieulafoy lesion is an uncommon disease which may lead to massive and repeated upper gastrointestinal hemorrhage. We report two cases of gastric Dieulafoy lesion successfully treated with either band ligation or endoscopic hemoclipping.

**Case presentation:**

*First case report:* A previously healthy 18-month-old female infant with *E. coli* sepsis, pneumonia and respiratory failure with bilateral pneumothorax requiring chest drainage. Over a few days, the patient presented hematemesis and melena with progressively worsening anemia. The esophagogastroduodenoscopy revealed an arterial vessel with eroded apex located between the body and the fundus of the stomach. Two elastic bands were applied which resulted in resolution of hematemesis and melena and improvement of the anemia.

*Second case report:* A 8-year-old male was admitted to our department with sudden massive hematemesis and melena. Clinical examination revealed anemia (hemoglobin, 6.8 g/dl). Esophagogastroduodenoscopy revealed a mucosal erosion with visible vessel located along the small curvature, close to the antrum. Three hemostatic clips were placed on the Dieulafoy lesion and hemostasis was obtained.

**Conclusions:**

we showed that, similar to gastric DL in adult patients,, gastric DL in pediatric patients can be successfully treated with endoscopic therapy, and both hemoclipping and band ligation are suitable techniques.

## Background

Dieulafoy lesion (DL) is an extremely rare cause of massive and repeated upper gastrointestinal (GI) bleeding in children, which may lead to consequences ranging from anemia to hypovolemic shock [1]. On microscopic examination, the lesion consists of the submucosal, abnormally large arterial vessel that has protruded through a 2-to-5 mm long mucosal defect. Bleeding starts from the small mucosal vessels and is followed by erosion of the vein. The artery tends to rupture soon afterward, resulting in massive bleeding [2]. The pathogenesis of vessel rupture and hemorrhage is uncertain but may be due to necrosis of the vessel wall induced by chronic gastritis [3].

DL is typically located in the fundus and in the small curvature of the stomach and only rarely in the distal part, with 80 to 95% of lesions occurring within 6 cm from the gastroesophageal junction. However, DL can be found along all the GI tract as well as in extra-GI sites such as the bronchial tree [4].

The incidence of DL in children remains undefined. DL has a male predominance (M:F 1.5:1) and it can occur at any age of life, starting from birth, which supports the proposed congenital origin of the disease [5].

Clinical features depend on the location of the lesions. Specifically, massive upper GI bleeding occurs with gastric and duodenal lesions. Small intestinal lesions lead to upper GI bleeding and hematochezia, while colonic lesions can cause fresh blood per rectum [6].

Currently, therapeutic endoscopy is the treatment method of choice for patients with acute active bleeding and it has substantially improved patients’ chances of survival, with the mortality rate decreasing from approximately 30–60% in the 1970s to 9-13% to date [7].

In this study, two cases of gastric DL successfully treated with two different endoscopic approaches are reported.

## Cases presentation

### First case report

A previously healthy 18-months-old female infant was referred to our hospital for *E. coli* sepsis, pneumonia and respiratory failure associated with bilateral pneumothorax requiring chest drainage. The child had no significant comorbidity. Blood examination at the admission showed elevated acute phase reactants, leukocytosis, and anemia (CRP 54.55 mg/L, PCT 4.75 ng/ml, WBC 18950/μl, Hb 8.8 g/dl). Then antibiotic therapy was administered. After an initial improvement of clinical status and laboratory parameters, a sudden deterioration of respiratory status was observed and intubation for assisted ventilation was started.

**Fig. 1:**
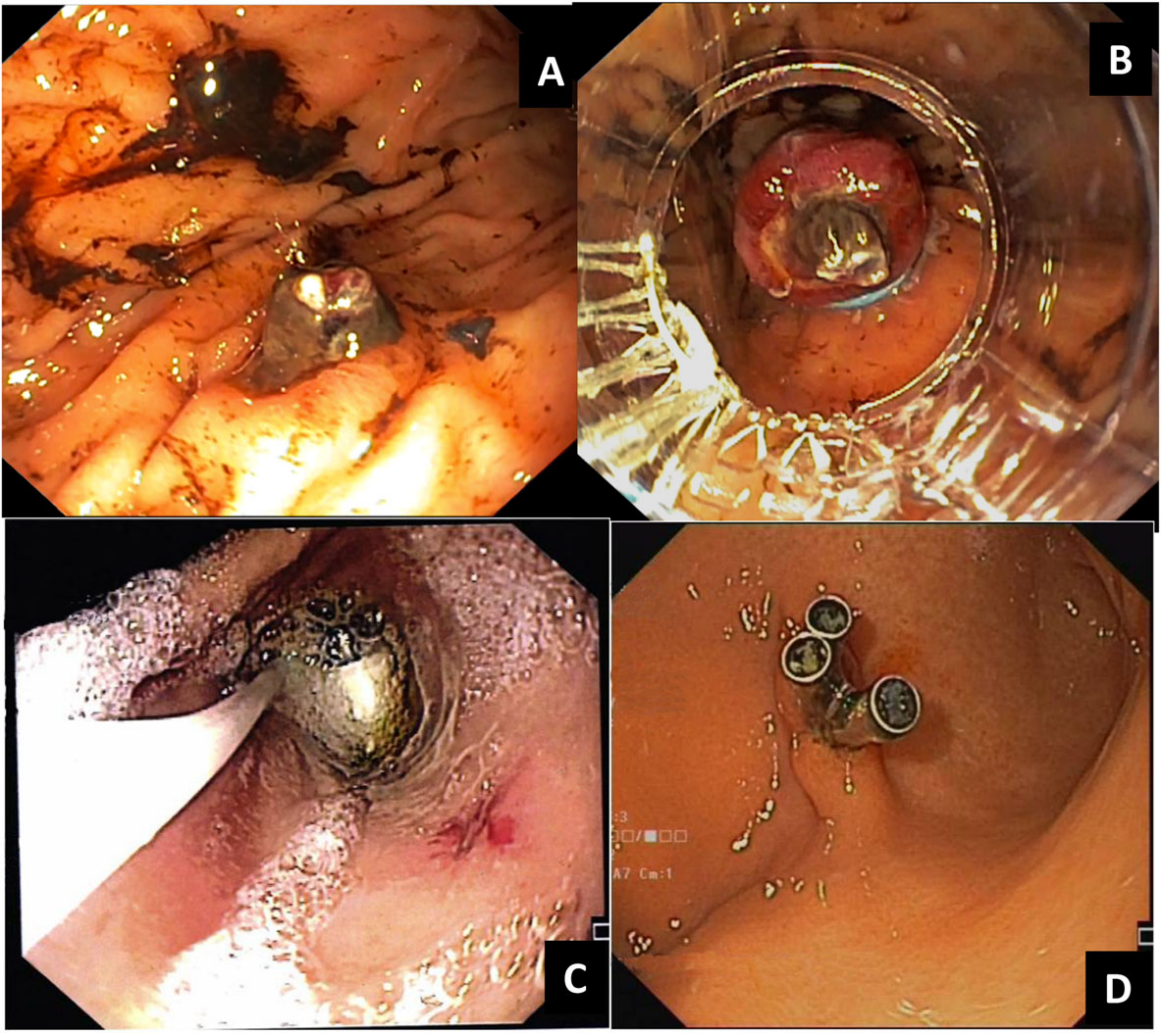
**a** arterial vessel with eroded apex in the large curvature of corpus; **b** band ligation with two elastics of the aberrant artery; **c** mucosal erosion with visible vessel in the small curvature of the antrum identified during capsule endoscopy placement; **d** endoscopic treatment with three hemoclips

Over a few days, the patient presented hematemesis and melena with progressively worsening anemia. EGDS revealed the presence voluminous clot in the gastric fundus. After its removal using a foreign body retrieval basket, an arterial vessel with eroded apex was identified in the large curvature, in the transition zone between the body and the fundus of the stomach (Fig. 1a). The diagnosis of DL were made and two elastic bandages were applied in order to perform band ligation of the aberrant artery, with consequent resolution of hematemesis and melena and improvement of the anemia (Fig. 1b).

### Second case report

An 8-years-old male was referred to our care for two episodes of hematemesis and melena. He had a four-day history of fever, vomiting, and diarrhea and he was being treated with antibiotic therapy. The child had no significant past medical illnesses, and he was healthy just before this episode. His family history was positive for colorectal cancer, heart disease and diabetes mellitus.

Clinical features suggested a hypotensive hemorrhagic shock and blood tests showed: Hb 6.8 g/dl, Ht 19.2%, MCV 82.1 fl, MCH 29.1 pg, albumin 2.0 g/dl, CRP 186 mg/L, PCT 33.3 ng/ml. A transfusion of packed RBCs and an infusion of albumin were performed. Meanwhile, EGDS and colonoscopy under general anesthesia were carried out.

The EGDS revealed several small erosions in the gastric body and fundus. At colonoscopy numerous clots without active bleeding were found. The day before, a video-capsule endoscopy was performed to evaluate the small intestine. During the endoscopic positioning of the video-capsule into the stomach, we identified the presence of mucosal erosion with visible vessel (Fig. 1c). It was located along the small curvature, close to the antrum. The diagnosis of DL were performed and treated with three endoscopic hemoclips (Fig. 1d). The images review of the video-capsule endoscopy was negative. The patient’s conditions subsequently improved, with the resolution of anemia.

After 5 months, the patient was admitted to our department for endoscopic re-evaluation. He was well at follow-up, with no recurrence of hematemesis or melena. Before the diagnostic procedure blood tests were carried out: Hb 13.2 g/dl, Ht 37%, MCV 82.9 FL, MCH 29.3 pg, ferritin 26 ng/ml and sideremia 84ugr/dl. The result of a blood coagulation test was also normal and the fecal occult blood was negative. The EGDS, under general anesthesia, showed the applied hemoclips, and no new gastric lesions with normal esophagus and duodenal mucosa were observed.

## Discussion and literature review

Diagnosis of the rare but potentially life-threatening DL are often delayed, even when recurrent GI bleeding has become clinically evident. Before the use of endoscopy as the first-line of choice for diagnosing DL, the diagnosis were basically made during autopsies or surgeries [8]. Currently, there is no consensus about the optimum treatment of DL; the best choice depends on clinical presentation, the site of the lesions and the physician’s own expertise in treating gastrointestinal bleedings.

Bleeding from DL was treated surgically before 1990 [9, 10]. Since then, endoscopic treatment has been recommended as first-line management for patients who are actively bleeding or for patients at risk of emergency surgery, recurrent bleeding, or mortality [11]. However, in a systematic revision of pediatric literature of the last 10 years, based on a total of five cases reports on pediatric gastric DL, one case was treated with surgery [12] and four cases received an endoscopic treatment. Details on gastric DL treated with endoscopic therapy are shown in Table 1 [13–16].

**Table 1:** Case reports of paediatric Dieulafoy lesion treated with endoscopic therapy since 2010

Authors	Age (sex)	Presentation	Location of DL	Comorbidity	Diagnostic tool	Treatment
**Coit et al. 2019** [13]	Neonate (M)	Haematemesis, melena	Stomach	Prematurity	GI endoscopy	Epinephrine injection
**Emura et al. 2016** [14]	2 months (M)	Haematemesis	Stomach	No	GI endoscopy	Haemoclip
**Chandane et al. 2016** [15]	6 months (M)	Haematemesis, melena, Haemorrhagic shock	Stomach	Enteric fever	GI endoscopy	Haemoclip
**Polonkai et al. 2011** [16]	Neonate (M)	Haematemesis	Stomach	Pyloric atresia	GI endoscopy	Haemoclip

Several advanced endoscopic techniques, including injections of epinephrine or sclerotherapy, mechanical interventions such as hemostatic clips and banding, and thermal treatments have been effective overall, even in the pediatric population [3, 17, 18].

Each technique has advantages and disadvantages in the treatment of DL. In particular, epinephrine is a safe and inexpensive method, but it is not recommended as monotherapy in DL due to the high re-bleeding rate [19]. Sclerotherapy using ethanol or polidocanol (sclerosing substances) can also be used to treat DL with good bleeding control [20]. Thermal endoscopic methods (i.e. bipolar and heater probes or Argon Plasma Coagulator) are not recommended because they carry a risk of transmural injury and could cause inadequate coagulation of the lesion [18].

To date, mechanical devices such as hemoclips and ligation bands are new choices for the treatment of GI bleedings. Hemostatic clipping has been found to be significantly more effective in preventing re-bleeding of gastric body DL than other methods. However, it is not easy to apply a hemoclip when the lesion is found in difficult sites such as the gastric fundus, the lower curvature of the stomach or the posterior wall of the duodenal bulb, or when the lesion has a fibrotic base. It is very important to have good expertise in this technique because a correct first application of hemoclips could prevent future needs of these [21, 22]. Band ligation is another mechanical hemostatic technique with very low perforation risk. It allows accessibility to difficult sites such as the esophagogastric junction and the posterior wall of the proximal body of the stomach. Occasionally, such as in the case of a residual vessel within a necrotic ulcer, band ligation may be associated with rare complications such as delayed bleeding [23]. Chung et al., compared the efficacy of hemostatic methods in patients with DL, and showed that mechanical methods for hemostasis, such as hemoclipping and band ligation, were superior to injection methods for controlling bleeding and preventing recurrent bleeding [24].

In this article, we confirm the efficacy of hemoclipping in the treatment of gastric DL and describe, for the first time, the use of banding in child. The choice between two different devices (banding or clipping) was performed according to the location of the DL, the diameter of visible vessel and on the endoscopist’s experience.

In conclusion, in our cases, we showed that, similar to DL in adults, gastric DLin children can be successfully treated with endoscopic therapy, and both hemoclipping and band ligation are suitable techniques.

## Data Availability

Not applicable.

## Abbreviations

DL: Dieulafoy lesions
GI: Gastrointestinal
EGDS: Esophagogastroduodenoscopy
